# Results From an RCT on Brief Parent Training: Long Term Effects on Parental Quality of Life

**DOI:** 10.3389/fpsyg.2019.00260

**Published:** 2019-02-18

**Authors:** Charlotte Reedtz, Sihu K. Klest, Nora Mortensen Aalo, Ingrid Daae Rasmussen, Joar Vitterso

**Affiliations:** Faculty of Health Sciences, UiT The Arctic University of Norway, Tromsø, Norway

**Keywords:** quality of life, the incredible years, short, parenting, parent training

## Abstract

**Background:** Previous research has shown that quality of life for adults decreases when they become parents, remains at a lower level than of non-parents and declines further with each child they have. Consistent with this, parents report that having children leads to more daily struggles and concerns than their work outside the home. In this study, we have investigated how participating in a brief parent training intervention influences parents’ quality of life. The aim of the study was to evaluate whether a brief, six-session version of an evidence-based parent training program (The Incredible Years), delivered in a non-clinical community sample, changed parent quality of life up to four years after the initial intervention.

**Methods:** Data were collected from mothers and fathers in a randomized controlled community trial (*N* = 117). Children’s mean age was 3.95 years at baseline, and 7.5 years at 4-year follow-up.

**Results:** There were no significant differences in the trajectory of change over the four time points between the intervention and control groups for mothers or fathers. However, results from analysing the linear change from pre to each of the other measurement points, revealed statistically significantly different change on life satisfaction after completing the intervention compared to the control group; immediately following the intervention, *t*(357) = 2.76, *p* = 0.006; and the difference between the groups was maintained three years after the intervention, *t*(360) = 3.14, *p* = 0.002.

**Conclusion:** The results of this study suggest that offering a parenting program focused on building a positive parent-child relation, has the potential to improve mothers’ quality of life. Implications of this are discussed.

**Clinical Trial Registration:** www.ClinicalTrials.gov, identifier NCT02850510. Retrospectively registered 29 July 2016.

## Introduction

“My children is the best thing that has ever happened to me” is a common saying among parents. However, previous research has found that parents are not necessarily more content or satisfied with their lives than non-parents (Dyrdal et al., 2010). Quality of life for adults decreases when they become parents and remains at a lower level than of non-parents until their children become legal adults (Rollins and Feldman, 1970; Dyrdal et al., 2010). In addition, parent satisfaction declines further with each child they have (Margolis and Myrskyla, 2011), and parents report that having children leads to more daily struggles and concerns than their work outside the home (Hansen et al., 2009).

A large study of 67,000 Norwegian mothers, conducted by Dyrdal and colleagues (Dyrdal et al., 2010) at the Norwegian Institute of Public Health (2010), followed women for a period of four years beginning in pregnancy. The results indicated that the maternal satisfaction declined dramatically when their baby is about 6 months old, and reached a low point during the child’s third year. One may wonder why becoming a parent poses such threat to maternal life quality, if the same is true for fathers, and what it would take to change this.

It seems evident that the marital/cohabitational relationship of parents may change in the transition to parenthood (Rollè et al., 2017), and that lack of partner support as well as conflict within the couple can predict stress, post-natal depressive and anxious symptoms in parents (Trillingsgaard et al., 2014). Furthermore, parenting stress and parental mental health is related to the dyadic adjustment between a child and its’ parents. Already two decades ago researchers established that maternal depression affected children’s social, behavioral, emotional and cognitive development negatively (Goodman and Gotlib, 1999). Recent research emphasize that the transmission of risk for psychopathology from parents to children is both diagnosis-specific and general and that the way parents cope with the parenting role and interact with their offspring is highly significant in this regard (Reedtz et al., 2019). In a recent study by Rollé and colleagues (Rollè et al., 2017) the researchers found that the level of parental distress, anxious and depressive symptoms appeared to be higher in mothers than in fathers. However, the results from their study showed that mental health mediated parenting stress and dyadic adjustment in both mothers and fathers during the first years of a child’s life.

Regardless of the reasons for the phenomenon that quality of life for adults’ drops after having children, it is evident that every day hassles and perceptions of reduced life quality after having children, may be risk factors for negative parenting practices. Furthermore, parent-child relations have a considerable impact on children’s mental, physical and social well-being. Numerous studies demonstrate that family risk factors such as harsh parenting practices (Reedtz et al., 2011a), family conflicts (Webster-Stratton, 1990), and divorce affect children’s short and long-term development (Sanders, 1999). Parents’ parenting practices in caring for their children influences the likelihood that children will develop socio-emotional and behavioral problems (Hutchings and Lane, 2005). More specifically, factors such as a lack of warm and positive interactions between parent and child, strict and inconsistent discipline, and parent psychopathology all increase the risk that children will develop emotional problems, behavior disorders, and physical health problems (Sanders, 1999; Shaw et al., 2000; Flaherty et al., 2013). Research has shown that parents’ child-rearing strategies, and the quality of care a child receives from her parents, are the strongest and most easily modified risk factors in the child’s development of behavioral and emotional problems (Morawska et al., 2009).

In addition to parents’ contributions to the development in their offspring, children also contribute to their own development though individual characteristics and functioning. For example, a child’s temperament has been emphasized as an important individual risk factor for psychiatric disorders later in childhood, also for disruptive behavior problems (Egger and Angold, 2006). Temperament represents individual differences in reactivity and regulation that are constitutional, present early in life, and relatively stable (Thomas and Chess, 1977), but also plastic to maturation and experience (Nigg, 2006). Temperamental traits related to aspects of attention, impulsiveness, and negative emotionality (frustration, intolerance, and being “hot-tempered”) are of special interest with regard to the development of disruptive behavior problems. In the case of such problems; the process of developing them may start with early characteristics of the child (i.e., neurobiological mechanisms of emotion regulation, temperament; Lewis et al., 2006), leading to differential responses from caregivers, which in turn contributes to social interaction patterns that lead to disruptive behavior problems (Snyder et al., 2003). In this perspective it makes sense that externalizing child behavior problems have been found to be negatively correlated with parents’ reports of satisfaction with their parenting role (Ohan et al., 2000). It is therefore important to offer preventive interventions to parents and children who are struggling with the parent-child relationship. Early parental intervention has been found to counter biological and environmental risk factors in children, creating a more positive developmental trajectory than would be expected without intervention (Watson et al., 2005; Morawska et al., 2009). Parenting programs provide helpful instruction to families seeking support in their communities. Cognitive-behavioral group-based parenting interventions are documented to be effective and cost-effective for improving disruptive behavior in childhood, parental mental health and parenting skills (Watson et al., 2005; Furlong et al., 2013). The Incredible Years (IY) series of parenting programs have shown to be effective both in Norway and in international studies in treating children with serious behavior problems (Hutchings and Lane, 2005; Foster et al., 2007; Drugli et al., 2009). In the present study, the long-term effects of a shortened version of the IY Basic Parenting Program (S-IY) in a non-clinical community sample on parents’ quality of life evaluated. The intervention aims to reduce risk factors related to development of child problem behaviors, support parents in strengthening their child’s social and emotional skills, teach positive parenting techniques, and reinforce parents’ competence. A previous RCT of the S-IY in the same sample as the present study, found a significant reduction in parents’ self-reported harsh parenting at post intervention and an increase in positive parenting and parents’ sense of competence for the group who received the S-IY program at one year and four year follow up assessments (Reedtz et al., 2011a; Scott et al., 2014).

However, it is interesting to study whether parents’ life quality could be altered by this brief parent training intervention. The aim of the present study was to evaluate whether the S-IY parenting program changed parent quality of life up to four years after the initial intervention for mothers and fathers. We investigated the difference between the intervention and control group on parents’ self-reported quality of life in their daily lives. We also evaluated differences in the trajectory of change between the intervention and control group. We hypothesized that the parents who received the S-IY would rate their quality of life higher than the control group.

## Materials and Methods

### Participants

A total of 269 families participated in the study. Of these 58 children (22%) were excluded from the study because they scored above the 90th percentile on the Eyberg Child Behavior Inventory (ECBI). The families who were excluded were offered the full 12–14 week IY basic program. Of the remaining 211 families, 22 (10%) dropped out during the first phase of the intervention.

**FIGURE 1:**
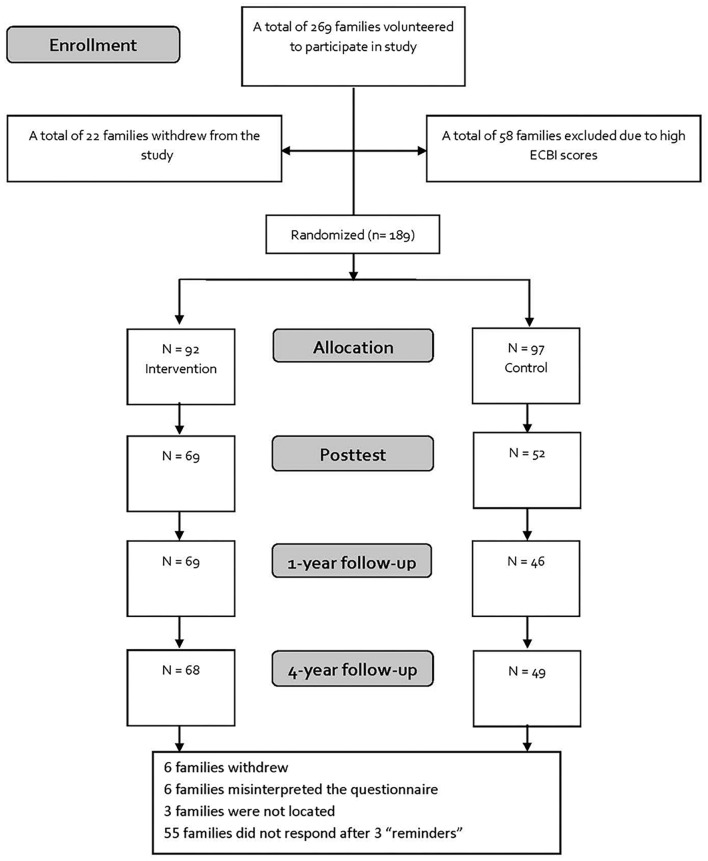
CONSORT flowchart.

The remaining families were randomly assigned to the S-IY intervention (*n* = 92) or to the control group where no intervention was given (*n* = 97). At baseline, the intervention and control groups were similar in demographic characteristics with no statistically significant differences between the two groups. They all agreed to complete assessments pre- and post-intervention, at 1-year post intervention follow-up, and 4-year follow-up. The families in the control group who received services as usual completed questionnaires on the same timeline as the intervention group. The response rates for post-test, one year follow-up, and four year follow-up were 75.3, 75.3, and 73%, respectively, for the intervention group and 53.6, 47.4, and 51% for the control group. Analyses of families who dropped out of the study at four year follow-up revealed that there were no statistically significant differences between families who completed the final assessment and those who did not on both demographic variables and all measures used in assessments. This was true both for analyses comparing the families who dropped out to those who remained in the study, as well as for analyses examining differential dropout between the control and intervention groups. The sample in the present study (Figure 1) consisted of parents participating on any measures after the pre-intervention assessment (*N* = 117).

The children ranged from 2 to 8 years of age; 112 boys (59%) and 77 girls (41%). The average age of the boys was 3.95 years (*SD* = 1.63) and girls 3.81 years (*SD* = 1.13) at T_1_. Mothers and fathers mean age at baseline was 35 and 37 years, respectively.

The parents in the sample worked full-time (61%), were couples/two adults in the household (80%) and had a bachelor’s degree or higher (78%) (Reedtz and Klest, 2016). The intervention and control groups were similar in demographic characteristics with no statistically significant differences between the two groups regarding child age, child gender, and other family characteristics.

### Measures

Parents answered a survey with questions about the child’s gender and age, the total number of children in the family, the birth order of the child, the parents’ age, marital status, employment status and education, and information on who had completed the questionnaire. Three standardized questionnaires were used to measure quality of life (QL).

#### Satisfaction With Life Scale (SWLS) (Reedtz et al., 2011b)

The SWLS was used to measure the parents’ general satisfaction with their lives. This scale captures cognitive satisfaction and gives the individual an opportunity to report how satisfied they are with their life. This five-item scale includes statements such as, “The relationships in my life are perfect” and “I am satisfied with my life.” Statements were answered on a Likert scale from 1 to 7 (1 = *strongly disagree* to 7 = *strongly agree*). Answers from the five items are summed into one scale with scores ranging from 5 to 35. The Cronbachs alpha in the present sample were α = 0.89.

#### Index of Well-Being (IWB) (Pavot and Diener, 1993)

The IWB scale was constructed to tap both cognitive and affective dimensions of general well-being, represented by two adjectives on each side of a 7-point scale. The instruction was to describe cognitions and experiences in life in terms of the adjective pairs defining each item. Examples of these adjectives are hopeless-hopeful, meaningful-meaningless, and hard to live-easy to live. Parents responded using the digits 1 to 7, which were listed between the adjectives defining each item. The Cronbachs alpha in the present sample were α = 0.91.

#### Flow-Simplex (FS) (Campbell et al., 1976)

The FS scale has two common items with the IWB scale includes four additional items, which taps perceptions and experiences of life. Examples of adjectives from the FS scale are interesting-uninteresting, pleasant-unpleasant, and lonely-social. Parents responded using the digits 1 to 7, which were listed between the adjectives defining each item. The Cronbachs alpha in the present sample were α = 0.86.

### Procedure

Families with children between the ages of 2–8 years in Tromsø, Norway were recruited through posters at day care and schools, advertisements in local newspapers, and invitations by mail. Tromsø is the largest city in northern Norway with a population of about 75,000. Interested families were informed about the project and received questionnaires. If there was more than one child between the age of 2 and 8, the youngest child was chosen as the target child in the study.

### Design

A randomized experimental control between-group design was used with pre- and post- intervention measurements, and at one-and four year follow-up. Children and families were randomized to either the shortened Basic version (*n* = 92), or the control group (*n* = 97). Families assigned to the control condition did not receive any intervention from the research project, but completed the questionnaires at the same time as the intervention groups.

### The Intervention

The parenting intervention, S-IY was shortened from the IY Basic parenting program (12–14 sessions) developed by Webster-Stratton (Vittersø, 2011), and is a manual- and video-based training program for parents of small children with behavior problems. The parents in the intervention group were divided into groups of 12–14 parents. Two group leaders led the program and, in the course of six weeks, the parents met weekly for two hours. The group leaders led discussions related to key aspects of parent training, using video clips, role-play and homework. The program taught parents positive parenting strategies (play, praise and reward), and the original manual for the first six sessions of the Basic IY program (Webster- Stratton and Hammond, 1997) was followed.

### Statistical Analyses

Version 24 of the statistical software package SPSS was used to conduct all analyses (IBM SPSS, Chicago, IL, United States).

Intention to Treat analysis (ITT) was used to incorporate every family who completed any questionnaire at the pre-intervention assessment. Estimated marginal means and standard errors are reported.

We used mixed models analysis [LMM; Multilevel analysis; (Singer and Willett, 2003)] in all of the analyses. In LMM we utilize the fact that data is hierarchical, where repeated observations (level 1) are nested within individuals (level 2). All observations in LMM are retained in the analysis through the use of full information maximum likelihood estimation (FIML). The FIML method of managing missing data introduces one of the lowest levels of bias to the results due to missing data (Graham, 2009). The analysis plan was as follows:

Stage (1) First we tested whether a linear change trajectory was sufficient or if a quadratic change trajectory would improve the model. For this evaluation we used a procedure suggested by Singer and Willett (Singer and Willett, 2003) comparing the –2^∗^log likelihood for two models, one with and one without the square of the time variable included in level 1 of the model.

Stage (2) Next we tested if the change pattern differed between the intervention and the control groups by assessing the significance of the time by group and/or time squared by group interaction. If quadratic time improved model fit in stage 1, time was represented by two variables, and consequently, effects concerning group differences over time were tested by assessing the combined effect of the time by group and time squared by group interactions using –2^∗^log likelihood comparisons for two models with and without these interaction terms.

Stage (3) Finally we tested for group differences for change contrasts between baseline and each of the three follow-up occasions.

In stage 1 and 2 time was represented as a continuous variable; the number of months since baseline, while in stage 3 time was treated as a categorical variable. Estimated marginal means were computed from the stage 3 model.

To evaluate statistical significance, a significance level of 0.05 was used.

## Results

Estimated marginal means and standard errors from baseline to each follow-up occasion are presented in Table 1.

**Table 1:** Estimated marginal means, standard errors.

	S-IY intervention				Control			
		
	T_1_	T_2_	T_3_	T_4_	T_1_	T_2_	T_3_	T_4_
	M (SE)	M (SE)	M (SE)	M (SE)	M (SE)	M (SE)	M (SE)	M (SE)
**Mothers**								
SWLS	23.66 (0.59)	25.84 (0.63)	26.20 (0.63)	26.11 (0.64)	24.87 (0.56)	24.89 (0.67)	25.82 (0.70)	24.74 (0.70)
IWB	43.33 (0.71)	45.63 (0.76)	45.41 (0.76)	45.00 (0.78)	44.71 (0.68)	44.60 (0.81)	44.90 (0.85)	44.73 (0.84)
FS	32.09 (0.50)	33.37 (0.54)	33.64 (0.54)	33.43 (0.55)	33.19 (0.48)	33.12 (0.57)	33.00 (0.60)	32.99 (0.59)
**Fathers**								
SWLS	24.07 (0.64)	24.66 (0.71)	25.02 (0.72)	24.36 (0.73)	25.92 (0.68)	25.75 (0.83)	27.18 (0,98)	27.32 (0.94)
IWB	41.88 (0.84)	42.32 (0.96)	43.87 (0.98)	42.57 (1.00)	46.55 (0.90)	45.09 (1.14)	47.21 (1.39)	47.31 (1.33)
FS	31.45 (0.60)	31.91 (0.68)	32.98 (0.69)	32.25 (0.71)	35.00 (0.64)	33.84 (0.81)	35.66 (0.97)	35.73 (0.93)


In the first stage of the analysis, we evaluated, for each of the three scales and for both informants, whether a quadratic change trajectory would significantly improve model fit over a linear change trajectory. For mother reported SWLS there was a significant improvement by adding the square of time to the model (change in –2^∗^log likelihood: Δχ^2^ (*df* = 4) = 23.5; *p* < 0.001). For the other five situations a quadratic model did not improve model fit, and hence a linear change trajectory was implemented.

### Satisfaction With Life Scale (SWLS)

#### Group Differences in the Change Trajectory

There were no significant differences in the trajectory of change over the four time points between the intervention and control groups for mothers, Δχ^2^ (*df* = 2) = 5.64; *p* = 0.06; Figure 1), or for fathers, Δχ^2^ (*df* = 1) = 1.09; *p* = 0.30).

**FIGURE 2:**
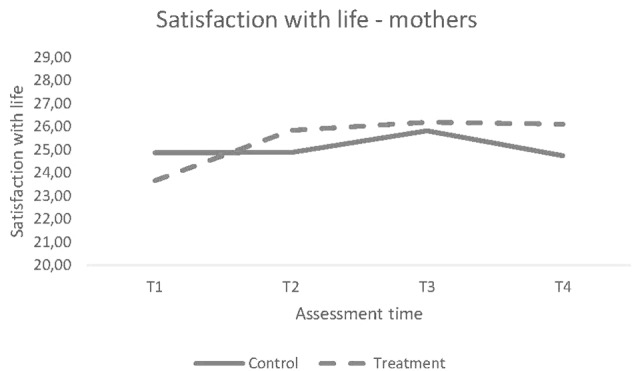
Trajectory of Change for Mothers’ scores on SWLS.

For mothers, the result indicate that there were no significant group differences for the SWLS scale both for the instantaneous rate of change at baseline and for the curvature of the quadratic change. In the case of fathers, we have no evidence of different linear change trajectories over the four time points (Figure 2).

#### Longitudinal Change From Baseline to Follow-Ups

To tap the nuances in the data material thoroughly, we analyzed the linear change from pre to each of the other measurement point as well. Analysis revealed statistically significant differences in change for mothers’ satisfaction with life between the S-IY and control groups from baseline to two of three post-intervention time points. The mothers in the S-IY group showed a significantly different change on life satisfaction after completing the intervention compared to the control group; immediately following the intervention (T2), *t*(357) = 2.76, *p* = 0.006; and the difference between the groups was maintained three years following completion of the S-IY program (T4), *t*(360) = 3.14, *p* = 0.002.

There were no statistically significant differences in change from baseline detected between the groups for fathers’ reports of life satisfaction at any follow-up time points using mixed model analysis, *F*(3, 193) = 0.89, *p* = 0.45.

### Index of Well-Being (IWB)

#### Group Differences in the Change Trajectory

There were no significant differences in the trajectory of change over the four time points between the intervention and control groups for mothers, Δχ^2^ (*df* = 1) = 0.77; *p* = 0.38), or for fathers, Δχ^2^ (*df* = 1) = 0.09; *p* = 0.76).

#### Longitudinal Change From Baseline to Follow-Ups

The mothers who received the S-IY group changed their scores significantly different on the IWB scale compared to the control group immediately after completing the intervention, *t*(355) = 2.52, *p* = 0.01. However, results were not significant for mothers one year following completion of S-IY, *t*(357) = 1.91, *p* = 0.06, and not at four year follow-up, *t*(358) = 1.66, *p* = 0.01.

The two groups of fathers did not differ on the outcome of the IWB scale in the mixed models analyses at any time point, *F*(3,201) = 0.72, *p* = 0.54.

### Flow-Simplex (FS)

#### Group Differences in the Change Trajectory

There were no significant differences in the trajectory of change over the four time points between the intervention and control groups for mothers, Δχ^2^ (*df* = 1) = 2.49; *p* = 0.11), or for fathers, Δχ^2^ (*df* = 1) = 0.10; *p* = 0.75).

#### Longitudinal Change From Baseline to Follow-Ups

**FIGURE 3:**
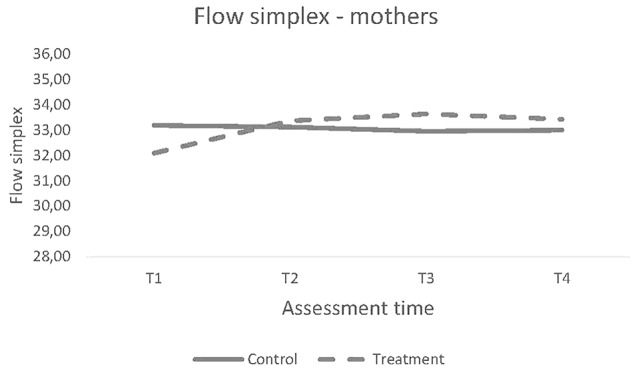
Trajectory of Change for Mothers’ scores on FS.

Even though the result was not significant, the mothers who participated in the S-IY program showed a strong tendency toward changing their scores different on the FS scale compared to the control group from baseline to post; *t*(360) = 1.95, *p* = 0.052, baseline to one year follow-up; *t*(362) = 2.47, *p* = 0.01, and baseline to four years follow-up; *t*(362) = 2.13, *p* = 0.03, following the completion of the intervention (Figure 3).

The two groups of fathers did not differ on the outcome of the FS scale in the mixed models analyses at any time point, *F*(3,199) = 0.93, *p* = 0.43.

## Discussion

The main findings of the present study were no significant differences in the trajectory of change over the four time points between the intervention and control groups for parents. However, we found significant increases in mothers’ satisfaction with life (SWLS), as well as a strong tendency for differential change on scores of dimensions of general well-being (FS), in mothers after having completed the S-IY program. Fathers did not experience significant changes in quality of life after the parent training.

The SWLS scores on the pre-test for both mothers and fathers, in both intervention and control groups, were relatively similar to the norm for SWSL in the general Norwegian population who score an average of 24 points (Dyrdal et al., 2010). Mixed models analyses showed that mothers in the S-IY group scored 1.22 points higher than those in the control group on SWLS after the intervention. Since satisfaction with life is a factor that generally remains relatively stable throughout life (Dyrdal et al., 2010), this finding is particularly notable. The scores for satisfaction with life among both mothers and fathers in intervention group were highest at 1-year follow-up. These findings are in line with previous research related to intervention effects in general, where the effects diminish somewhat over time (Fixsen et al., 2005).

There may be several reasons for the mothers’ reported increase in quality of life after having completed the S-IY program. Parents experiencing an increased level of competence in the parenting role may experience better quality of life (Reedtz and Klest, 2016). It is likely that the balance between the parents’ feelings of burden related to child rearing, and the resources they have to cope with child-rearing challenges, is reflected in the parents’ ability to handle daily routines. Researchers have postulated that there are four factors that predict how burdensome and stressful a situation is perceived; namely, degree of importance, overload, degree of ambiguity, and the degree to which the situation cannot be controlled (Ogden, 2007). Being a good parent and caring for one’s offspring in the best way possible is of great importance for most parents. Parents with small children may also feel overwhelmed. Dealing with the demands of toddlers and their needs are demanding for most parents, and family conflicts may occur as a result. Children are not born with instructions, as were stated on the recruitment posters for this study. For most parents of small children, it may take some time before normal challenges in parent-child interactions are addressed in proper ways. This may result from the ambiguity of where the problem lies, and parents who do not have adequate means to manage behavior problems in their children can experience the situation as uncontrollable. In the S-IY program, parents learn concrete child-rearing strategies that they can use in their daily interactions with their children. This may help the parents feel more competent, more satisfied, to experience less stress in their everyday lives, and to experience less conflict-ridden family interactions.

A meta-analytic review from 2003 (Twenge et al., 2003) that examined 100 studies on the transition to the parenting role, revealed that dissatisfaction surrounding the parenting role is greater the higher up the parents find themselves on the socioeconomic ladder. It is conceivable that this may stem from the fact that these parents feel they have made a greater sacrifice in their own lives in relation to constraints on autonomy, finances and opportunities for career development, as compared to those who have lower socioeconomic status. The strengthened satisfaction with life post intervention for mothers in this study is very interesting in this perspective, since most of the parents who participated had a higher educational level than the general population (Reedtz and Klest, 2016).

Furthermore, in the same review (Twenge et al., 2003) mothers of infants were also found to be more dissatisfied compared to mothers of older children and fathers of all children. These results suggested that the decrease in satisfaction was greatest for those parents who had the largest responsibility for the child. This may serve as a possible explanation for the finding that fathers in the present study did not experience strengthened quality of life after the intervention. Even though there has been a development toward more equality between the sexes in Norway, mothers still have more responsibility for young children and household chores compared to fathers’ (Statistics Norway, 2018). In other words, being a father does not constraint the autonomy and freedom of fathers as much as for mothers, neither related to work nor home activities. There still may be some truth in the common view that success in a woman’s life is closely related to being a good mother, whereas success in a man’s life is not so as closely related to parenthood.

## Limitations

The most important limitation of the present study is that characteristics of the children in the study; such as child behaviors, and temperament, are not measured. Neither are variables such as couple relationship and other parental risk factors. Such data would have provided interesting information and constituted the basis for more exhaustive statistical analyses on what determines the quality of life in parents.

## Conclusion

Having children is often described as one of the great joys experienced by adults. However, parenting can also be very demanding and it is common for parents to experience a reduced quality of life after having a child. The results of this study suggest that offering a preventive and positively focused parenting program (S-IY) has the potential to improve mothers’ quality of life. These are especially promising results because life satisfaction is generally a stable trait that is difficult to improve. The IY parenting program was in this study because it has been implemented throughout Norway, and has an existing framework for successful implementation with many trained practitioners. However, other parenting interventions could also prove to offer relevant support and hence to strengthen their quality of life. Future research should investigate the relationship between parent practices, parents’ sense of competence and their quality of life, in addition to identifying the effect parents’ perceived quality of life has on their children’s short and long-term outcomes.

## Data Availability

The datasets used and/or analyzed during the current study are available from the corresponding author on reasonable request.

## Ethics Statement

The Regional Committees for Medical and Health Research Ethics (REC) in Norway approved the study. This was done by the REC North secretariat, UiT the Arctic University of Norway, reference 2016/1532. All participants gave informed and written consent to the study.

## Author Contributions

NA, IR, CR, and SK analyzed and interpreted the parent data. CR and SK were major contributors in writing the manuscript. All authors read and approved the final manuscript.

## Conflict of Interest Statement

The authors declare that the research was conducted in the absence of any commercial or financial relationships that could be construed as a potential conflict of interest.
